# Inflammation and Long-Term Mental Health Outcomes in Post-COVID-19 Patients: A Follow-Up Study

**DOI:** 10.1192/j.eurpsy.2026.11020

**Published:** 2026-07-31

**Authors:** A. Duque Guerra, J. Pérez-Blanco, E. Grasa, G. Riquelme, F. Vidal-Domènech, M. Sanmartí, V. Diaz-Brito, A. Barajas, J. López-Contreras, B. Ramos

**Affiliations:** 1 Dept. Psychiatry, Hospital de la Santa Creu i Sant Pau, Barcelona, Spain; 2 Centro de Investigación Biomédica en Red de Salud Mental (CIBERSAM), Institut de Recerca Biomèdica Sant Pau (IIB-Sant Pau), Barcelona, Spain; 3 Psiquiatria Molecular, Parc Sanitari Sant Joan de Déu, Institut de Recerca Sant Joan de Déu, Dr. Antoni Pujadas, 42, 08830 Sant Boi de Llobregat, Spain; 4 Servicio de Enfermedades Infecciosas, Parc Sanitari Sant Joan de Déu, Institut de Recerca Sant Joan de Déu, Dr. Antoni Pujadas, 42, 08830 Sant Boi de Llobregat, Spain; 5 Servicio de Enfermedades Infecciosas, Hospital Universitari Parc Taulí, Institut d’Investigació i Innovació Parc Taulí (I3PT-CERCA), Universitat Autònoma de Barcelona, Sabadell, España; 6 Departamento de Psicología Clínica y de la Salud, Universitat Autònoma de Barcelona, Cerdanyola del Vallès, Barcelona, España; 7 Serra Húnter Programme. Generalitat de Catalunya, Barcelona, España; 8 Unitat de Malalties Infeccioses, Hospital de la Santa Creu i Sant Pau, Barcelona, España; 9 Centro de Investigación Biomédica en Red de Salud Mental (CIBERSAM), Instituto de Salud Carlos III, Madrid, España; 10 Facultad de Medicina. Universidad de Vic- Universidad Central de Cataluña, Vic, España., Barcelona, Spain

## Abstract

**Introduction:**

The COVID-19 pandemic has been associated with increased rates of depression, anxiety, and post-traumatic stress disorder (PTSD). While systemic inflammation during acute infection has been proposed as a biological mechanism, its relationship with long-term mental health outcomes remains unclear. This study investigates psychiatric symptoms and their association with inflammation and illness severity between 24 and 36 months after COVID-19.

**Objectives:**

To evaluate the prevalence of psychiatric symptoms (depression, psychological distress, and post-traumatic symptoms) 24–36 months after COVID-19 infection.

To examine the association between inflammatory markers (measured during the acute phase and at long-term follow-up) and psychiatric outcomes.

To explore the relationship between COVID-19 severity and psychiatric symptoms.

**Methods:**

A multicenter observational cohort study enrolled 92 Spanish patients with confirmed SARS-CoV-2 infection. Inflammatory markers (C-reactive protein levels, neutrophil-to-lymphocyte ratio, systemic immune-inflammation index, platelet counts) were measured during acute illness and reassessed between 24 and 36 months after infection. Psychiatric symptoms at two years were assessed using the Beck Depression Inventory-II (BDI-II), Symptom Checklist-90-Revised (SCL-90-R) for psychological distress, and Davidson Trauma Scale (DTS). COVID-19 severity was classified according to WHO criteria.

**Results:**

15% of patients showed mild depression level, 21 % of the patients had post-traumatic symptoms, while 28% of patients had psychological distress with somatization and obsessive-compulsive symptoms being most prevalent. Platelet counts during the acute phase were the only inflammatory parameter significantly associated with higher SCL-90-R scores for somatic and anxiety-related psychological distress. No significant associations were found between inflammatory markers—either during the acute phase or at follow-up—and psychiatric symptoms measured by the BDI-II and DTS. COVID-19 severity was correlated with increased obsessive–compulsive, somatic, and anxiety-related symptoms on the SCL-90-R.

**Conclusions:**

Between 15% and 28% of patients presented psychiatric symptoms, with psychological distress being the most consistent finding. Furthermore, obsessive–compulsive, somatic, and anxiety-related symptoms were associated with COVID-19 severity. These findings highlight the need for ongoing psychological monitoring and comprehensive approaches to understanding and supporting post-COVID mental health.

**Disclosure of Interest:**

None Declared

